# Plasma, urine and ligament tissue metabolite profiling reveals potential biomarkers of ankylosing spondylitis using NMR-based metabolic profiles

**DOI:** 10.1186/s13075-016-1139-2

**Published:** 2016-10-22

**Authors:** Wei Wang, Gen-jin Yang, Ju Zhang, Chen Chen, Zhen-yu Jia, Jia Li, Wei-dong Xu

**Affiliations:** 1 Department of Orthopedics, Chengdu Military General Hospital, Chengdu city, People’s Republic of China; 2 School of Pharmacy, Second Military Medical University, Shanghai city, People’s Republic of China; 3 Department of Rheumatology, Changhai Hospital, Shanghai city, People’s Republic of China; 4 Physical Examination Center, Changhai Hospital, Shanghai city, People’s Republic of China; 5 Department of Orthopedics, Changhai Hospital, Shanghai city, People’s Republic of China

**Keywords:** Ankylosing spondylitis, NMR, Biomarkers, Metabolomics

## Abstract

**Background:**

Ankylosing spondylitis (AS) is an autoimmune rheumatic disease mostly affecting the axial skeleton. Currently, anti-tumour necrosis factor α (anti-TNF-α) represents an effective treatment for AS that may delay the progression of the disease and alleviate the symptoms if the diagnosis can be made early. Unfortunately, effective diagnostic biomarkers for AS are still lacking; therefore, most patients with AS do not receive timely and effective treatment. The intent of this study was to determine several key metabolites as potential biomarkers of AS using metabolomic methods to facilitate the early diagnosis of AS.

**Methods:**

First, we collected samples of plasma, urine, and ligament tissue around the hip joint from AS and control groups. The samples were examined by nuclear magnetic resonance spectrometry, and multivariate data analysis was performed to find metabolites that differed between the groups. Subsequently, according to the correlation coefficients, variable importance for the projection (VIP) and *P* values of the metabolites obtained in the multivariate data analysis, the most crucial metabolites were selected as potential biomarkers of AS. Finally, metabolic pathways involving the potential biomarkers were determined using the Kyoto Encyclopedia of Genes and Genomes (KEGG) database, and the metabolic pathway map was drawn.

**Results:**

Forty-four patients with AS agreed to provide plasma and urine samples, and 30 provided ligament tissue samples. An equal number of volunteers were recruited for the control group. Multidimensional statistical analysis suggested significant differences between the patients with AS and control subjects, and the models exhibited good discrimination and predictive ability. A total of 20 different metabolites ultimately met the requirements for potential biomarkers. According to KEGG analysis, these marker metabolites were primarily related to fat metabolism, intestinal microbial metabolism, glucose metabolism and choline metabolism pathways, and they were also probably associated with immune regulation.

**Conclusions:**

Our work demonstrates that the potential biomarkers that were identified appeared to have diagnostic value for AS and deserve to be further investigated. In addition, this work also suggests that the metabolomic profiling approach is a promising screening tool for the diagnosis of patients with AS.

## Background

Ankylosing spondylitis (AS) is a form of chronic inflammatory arthritis that predominantly affects the axial skeleton causing patients to experience severe stiffness and pain [1]. In addition, AS affects the peripheral joints and is exhibited by extra-articular symptoms such as inflammatory bowel disease (IBD) and uveitis [2]. Because early AS is often asymptomatic or does not show obvious pathological changes, the diagnosis of AS is not prompt. Although the significance of early intervention has been well-recognized for a long time, the diagnostic criteria for early-stage AS may be difficult to apply, leading to delay in the use of appropriate intervention [3].

Currently diagnosing AS is a clinically driven process based on the observation of clinical symptoms and structural changes on x-rays. The structural changes on x-rays are the result of the inflammatory process but do not make the inflammatory process itself known [4]. It takes several years before the changes are visible on x-rays. Diagnostic biomarkers such as rheumatoid factor or autoantibodies against citrullinated proteins are used for the early diagnosis of rheumatoid arthritis (RA) with high specificity and selectivity [5, 6]. However, potential markers for the diagnosis of AS, such as immunoglobulin G (IgG), IgA or C-reactive protein, have not achieved sufficient diagnostic sensitivity or specificity [7]. Additionally, the high association of human leucocyte antigen B27 with AS, combined with a relatively high population prevalence, does not make it a good candidate for use as a diagnostic marker alone, but it may be useful in a combined model [4].

With the advent of microarray techniques many pathological processes can be explored and monitored globally with the aid of omics-driven, high-throughput technologies [8]. Genomics, transcriptomics and proteomics have emerged as biochemical profiling tools to provide important insight into the biology of various diseases [9]. However, these profiling methods are focused only on upstream genetic and protein variations [10]. As a rapidly developing field of systems biology, metabolomics represents a new method that delineates a wide panel of metabolic parameters and thus allows a global and potentially more personalized diagnostic method to be used in combination with conventional protocols [11]. The fundamental basis for the application of clinical metabolomics is that perturbations are in biological [12]. To date, metabolic profiling has been used to identify potential biomarkers for other arthritic diseases, including RA [13], osteoarthritis [14] and gout [15].

Several research studies but not many, have used metabolomics in the study of AS. For example, Fischer et al. found that vitamin D_3_ metabolites were down-regulated in AS [16]. Gao et al. found that the plasma concentrations of some amino acids were abnormally changed in AS [8]. In addition, Chen et al. found that some plasma fatty acid chains in AS could be used as potential biomarkers [1]. All of the research described above used only plasma as a test sample and mass spectrometry (MS) as a detection method.

The two most powerful and most commonly used analytical methods for metabolic fingerprinting are MS and nuclear magnetic resonance (NMR) spectrometry [17]. NMR is a non-invasive and non-destructive technique that can provide complete structural analysis of a wide range of organic molecules in complex mixtures [18]. It has a series of advantages compared with MS including simple sample preparation, not requiring chromatographic separation and being inexpensive on the basis of consumables [19]. Although a growing number of NMR-based metabolomic studies are aimed at finding potential biomarkers of several diseases, such as RA [13], prostate cancer [20] and diabetes [21], there have been no reports of using an NMR method to investigate the differences of metabolites in AS. Urine and plasma are the most frequently used specimens for exploring the systematic alteration of metabolites in humans because the collection and handling of these specimens are relatively easy [22]. Furthermore, pathologic tissues have been used as samples for metabolic profiling in a growing number of studies because these tissues can provide more metabolic information directly related to these diseases [19, 20]. However, thus far, only plasma has been used as a research sample for metabolomic studies of AS, and the results may not be comprehensive.

In the present study using NMR spectroscopy, we performed metabolic profiling to observe the metabolites in plasma and urine samples of patients with AS and healthy control subjects. Furthermore, to provide complementary information on metabolites in intact tissues, ligament tissues surrounding hip joints of patients with AS who were scheduled for hip arthroplasty were collected for metabolomic analysis. The hip joint is the most easily involved peripheral joint of AS, and ossification of the ligament is the most important pathological change [23], these are the main reasons why this ligament tissue was chosen as the test sample. To the best of our knowledge, this study is the first in which NMR has been used as a detection method and in which biological samples other than plasma have been used as test samples to perform a metabolomic study of AS to identify more suitable potential biomarkers.

## Methods

### Patients

Volunteers were recruited mainly from among patients with AS who were admitted to the rheumatology and immunology clinic of our hospital (Changhai Hospital, Shanghai, China) to participate in plasma and urine analyses in this study between June 2014 and June 2015. Volunteers were also recruited for the sample analysis of ligament tissues surrounding hip joints of patients with AS who were scheduled for hip arthroplasty in the department of orthopaedics of our hospital between June 2013 and June 2015. The inclusion criteria for recruitment were as follows: aged >18 years with an independent right to sign a consent form and with a diagnosis of AS (1984 New York modified criteria) [24]. The exclusion criteria were as follows: patients with other systemic diseases (hypertension, diabetes and the comorbidities of AS), patients with a history of taking medication within the last week (e.g., treated with anti-tumour necrosis factor α [anti-TNF-α], taking disease-modifying anti-rheumatic drugs or non-steroidal anti-inflammatory drugs), and patients who had breakfast in the morning before they came to the outpatient clinic (for plasma and urine samples). Additionally, age- and sex-matched volunteers were recruited from among the healthy population for routine physical examinations in our hospital as control subjects for plasma and urine sample analysis, and their exclusion criteria were the same as those for the AS group. Volunteers were also recruited from among patients admitted to the emergency department of our hospital for the surgical treatment of femoral neck fracture (FNF) as control subjects for ligament tissue sample analysis (no other underlying diseases were allowed for these patients to be included). All procedures performed in this study involving human participants were carried out in accordance with the 1964 Helsinki declaration and its later amendments or comparable ethical standards. This clinical study was approved by the ethics committee of Changhai Hospital (CHEC2013-176).

### Sample collection and processing

Urine and plasma samples were collected from all of the fasting volunteers in the morning. Blood samples were first placed in heparin anti-coagulation tubes at room temperature and then centrifuged at 4 °C (5000 rpm for 10 minutes) [25]. Next the supernatant plasma was divided into aliquots of 300 μl in epoxy epoxide (EP) tubes and stored at −80 °C for later detection and analysis. Urine samples were collected and then centrifuged at 4 °C within 30 minutes (6000 rpm for 15 minutes). After that step, the supernatant was divided into aliquots of 540 μl in EP tubes and stored at −80 °C for later detection and analysis [26].

Samples of ligament tissue surrounding the hip joints were collected from the volunteers during hip surgery. In the operating theatre, the ligament tissue was cut into small pieces of 1 cm^3^ each, aliquoted in cryogenic tubes and then stored in a liquid nitrogen tank [23]. The liquid nitrogen tank was brought to the laboratory, and the tissue was immediately homogenized into powder using an electric tissue homogenizer (60 Hz for 40 seconds). The powder was removed into an EP tube and weighed. Then, extract solution (2.85 ml/g distilled water + 4 ml/g methanol and chloroform) was added. Samples were vortexed for 15 seconds three times and kept on ice in between. Last, samples were centrifuged at 4 °C (10,000 × *g* for 10 minutes). Three hundred microliters of each supernatant (polar extracts) and subnatant (non-polar extracts) were removed into two EP tubes. Dried products of the supernatant and subnatant were obtained using freeze-drying treatment and a nitrogen blowing instrument, respectively, and they were stored at −80 °C for detection and analysis [27].

### Sample preparation and spectroscopy

The plasma samples were prepared for NMR analysis by mixing 300 μl of plasma with 300 μl of PBS (1.5 M NaH_2_PO_4_/K_2_HPO_4_ pH 7.4, 10 % vol/vol D_2_O) containing 0.6 mg of 3-trimethylsilyl-2,2,3,3-d_4_-propionate (TMSP) as a chemical shift reference (δ = 0.00 ppm). Urine samples (540 μl mixed with 60 μl of PBS) and tissue extracts (mixed with 600 μl of PBS) were processed similarly. All of the samples were then centrifuged at 4 °C (12,000 rpm for 10 minutes), and the supernatants were pipetted into 5-mm NMR tubes for NMR analysis [27].

The proton NMR spectra of the plasma, urine and tissue extract samples were recorded at 300 K on a Bruker Avance II 600-MHz spectrometer (Bruker BioSpin, Rheinstetten, Germany) operating at a ^1^H frequency of 600.13 MHz and equipped with a broadband observe probe.

Standard water-suppressed one-dimensional spectra of urine and tissue extracts were acquired using the first increment of the gradient-selected nuclear Overhauser effect spectroscopy pulse sequence (recycle delay-90 degrees-*t*_1_-90 degrees-*t*_m_-90 degrees-acquire data) with a recycle delay of 2 seconds, a *t*_1_ of 3 microseconds, a mixing time (*t*_m_) of 100 milliseconds and a 90-degree pulse length of 13.70 microseconds. A total of 128 transients were acquired in 49,187 data points using a spectral width of 9590 Hz and an acquisition time of 2.56 seconds. For plasma, a water-presaturated Carr-Purcell-Meiboom-Gill pulse sequence (recycle delay-90 degrees-(τ-180 degrees-τ)_n_-acquisition) was employed to attenuate the NMR signals from macromolecules. A spin-spin relaxation delay (2*n*τ) of 76.8 milliseconds and a spin-echo delay τ of 400 microseconds were used. Typically, the 90-degree pulse was set to 13.7 microseconds, and 32 transients were collected in 49,178 data points for each spectrum using a spectral width of 9590 Hz. Other acquisition parameters were the same as described above [28]. After the Fourier transformation, phase correction and baseline correction were performed using the TopSpin version 3.0 software package (Bruker BioSpin). The ^1^H chemical shifts were referenced to the TMSP peak at δ = 0.00.

### Multivariate data analysis

Multivariate statistical analysis can take internal relationships and mutual influence between the variables into account; therefore, the use of multivariate statistical analysis was more reasonable than univariate statistical analysis with respect to the data source, with many objectively existing variables and mutual influences. Multivariate statistical analysis, including principal component analysis (PCA), partial least squares discriminant analysis (PLS-DA) and orthogonal projection to latent structures discriminant analysis (OPLS-DA), was performed to globally understand the metabolic changes of AS.

PCA was performed using the SIMCA-P version 11.5 software package (Umetrics AB Umea, Sweden). PCA is an unsupervised analytical pattern recognition tool that provides an overview of complex data through examination of the covariance structure. The multivariate data can be displayed in a few principal components as a set of ‘scores’ that highlight general trends and outliers [29].

PLS-DA and OPLS-DA were performed using a unit variance scaled approach based on the SIMCA-P version 11.5 software package (Umetrics AB). PLS-DA is a supervised regression method used to maximize the covariance between the predictor space and the response space. It can predict responses in the population using the predictor matrix. OPLS-DA was performed with the NMR data to facilitate interpretation of the loading. The model coefficients were back-calculated from the coefficients incorporating the weight of the variables and plotted with color-coded coefficients to enhance the interpretability of the model [29]. The color-coded correlation coefficients indicate the significance of the metabolite contribution in predicting the response. Two parameters, *R* ^2^ *Y* and *Q* ^2^, were used for evaluation of the models. *R* ^2^ *Y* explains the latent variables of the sums of squares of all *x* and *y* values. *Q* ^2^ reflects the cumulative cross-validated percentage of the total variation that can be predicted by the current latent variables. High coefficient values of *R* ^2^ *Y* and *Q* ^2^ *Y* (>0.5 is acceptable) showed good discrimination and predictive ability [8]. In the PLS-DA models, we used permutation tests (200 times) to observe whether there was overfitting, while the permutation test was evaluated by using cross-validation, with *R* ^2^ and *Q* ^2^ as correlation coefficients of the cross-validation. It is generally believed that the intercept of the *Q* ^2^ regression line on the *y*-axis (permutation plot) being 0 or less than 0 indicates a reliable and effective model, without overfitting [30].

### Potential biomarker selection and metabolic pathway analysis

On the basis of parameters obtained from the multivariate analysis, we selected the metabolites whose absolute correlation coefficient values greater than the cut-off value or variable importance for projection (VIP) (value >1.0 and *P* < 0.05) as potential biomarkers for AS [10, 29, 30]. Then, the related metabolic pathways in which these potential biomarkers are involved were identified through a Kyoto Encyclopedia of Genes and Genomes (KEGG) database retrieval, and the metabolic pathway map was plotted.

### Statistical methods

IBM SPSS version 20.0 software (IBM Armonk, NY, USA) was used for statistical analysis. The mean values for age, body mass index (BMI), disease duration, Bath Ankylosing Spondylitis Disease Activity Index score (BASDAI) and Bath Ankylosing Spondylitis Functional Index score (BASFI) were reported with their SDs. A *P* value ≤0.05 was considered to be statistically significant.

## Results

### Clinical population

Upon our invitation, 44 patients with AS and 44 healthy individuals as the control group consented to participate in the study of plasma and urine samples, and another 30 patients with AS and 30 patients with FNF consented to participate in the study of tissue samples. All of the participants met our inclusion or exclusion criteria, and 5 patients with AS (6.3 %) with hypertension, diabetes mellitus and AS comorbidities (iritis, ulcerative colitis and otitis media) were excluded. The clinical information of the patients and control subjects is summarized in Table 1. As listed in the table, the age, sex and BMI of the control group basically matched those of the AS group.

**Table 1:** Participant characteristics at the time of sampling

Characteristics	Urine and plasma samples			Ligament tissue samples		
	Patients with AS	HC	*P* value^a^	patients with AS	Patients with FNF	*P* value^a^
Number of participants	44	44	–	30	30	–
Age, years, mean ± SD	31.8 ± 10.9	33.8 ± 9.7	0.350	40.6 ± 12.8	46.6 ± 11.6	0.065
Range	18–59	18–57	–	20–71	31–72	–
18–29	15	12	–	6	2	–
30–49	21	22	–	14	13	–
50–79	8	10	–	10	15	–
Sex, *n* (%)			1.000			0.037
Female	6 (13.6 %)	6 (13.6 %)	–	4 (13.3 %)	11 (36.7 %)	–
Male	38 (86.4 %)	38 (86.4 %)	–	26 (86.7 %)	19 (63.3 %)	–
BMI, kg/m^2^, mean ± SD	21.8 ± 2.4	22.5 ± 2.9	0.226	22.8 ± 2.0	23.7 ± 1.5	0.062
<18.5	13	12	–	9	7	–
18.5–25	25	23	–	14	15	–
>25	6	9	–	7	8	–
Disease duration, years, mean ± SD	6.8 ± 3.5	–	–	14.2 ± 4.8	–	–
<5	25	–	–	0	–	–
5–10	18	–	–	11	–	–
>10	1	–	–	19	–	–
BASDAI score	3.2 ± 1.8	–	–	5.8 ± 1.1	–	–
0–3	20	–	–	0	–	–
3–6	23	–	–	18	–	–
6–10	1	–	–	12	–	–
BASFI score	3.9 ± 2.1	–	–	5.5 ± 0.9	–	–
0–3	20	–	–	0	–	–
3–6	22	–	–	19	–	–
6–10	2	–	–	11	–	–
Treatment duration, years, mean ± SD	2.8 ± 1.5	–	–	8.4 ± 3.6	–	–
0	32	–	–	0	–	–
1–10	11	–	–	19	–	–
11–20	1	–	–	9	–	–
>20	0	–	–	2	–	–

*Abbreviations: AS* Ankylosing spondylitis, *FNF* Femoral neck fractures, *BMI* Body mass index, *BASDAI* Bath Ankylosing Spondylitis Disease Activity Index, *BASFI* Bath Ankylosing Spondylitis Functional Index, HC Healthy control subjects

^a^Calculated using Student’s *t* test for continuous variables and chi-square test for categorical variables between patients with AS and healthy control subjects

### ^1^H NMR spectra of samples

**Fig. 1:**
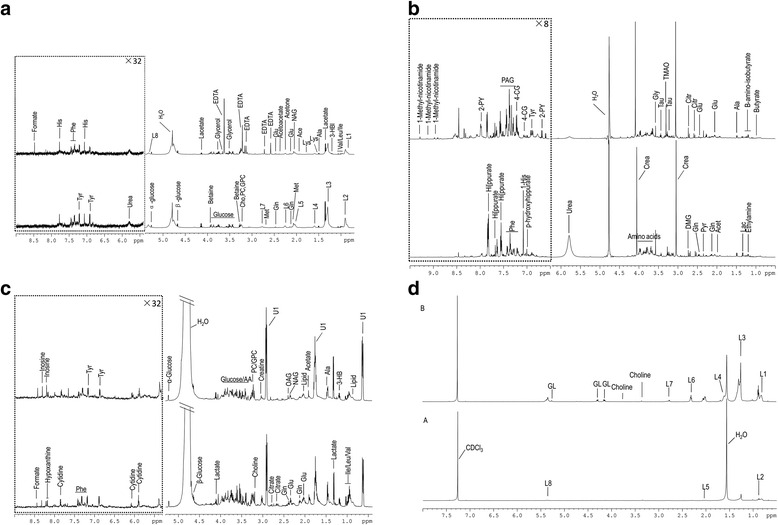
Representative 600-MHz ^1^H NMR spectra of (**a**) plasma, (**b**) urine and (**c**) polar tissue extract and (**d**) non-polar extract metabolites obtained from the AS group (*upper layer*) and control subjects (*lower layer*). *Phe* Phenylalanine, *His* Histidine, *Glu* Glutamate, *Ace* Acetate, *Lys* Lysine, *Ala* Alanine, *Val* Valine, *Leu* Leucine, *Ile* Isoleucine, *Tyr* Tyrosine, *Met* Methionine, *Gln* Glutamine, *PC* Phosphorylcholine, *GPC* Glycerophosphocholine, *OAG* O-acetyl glycoprotein, *Lac* Lactate, *Pyr* Pyruvate, *Citr* Citrate, *Gly* Glycine, *4-CG* 4-Cresol glucuronide, *GL* Glyceryl of lipids, *3-HB* 3-Hydroxybutyrate, *NAG* N-acetyl glycoprotein, *NMR* Nuclear magnetic resonance, *TG* triglycerides, *PAG* phenylacetylglycine, *2-PY* 2-Pyridone-3-carboxamide, *L1*:C***H***_*3*_ *(CH*_*2*_ *)*_*n*_., *L2* C***H***_3_CH_2_CH_2_C=, *L3* -(C***H***_2_)_n_-, *L4* C***H***_2_CH_2_CO, *L5* -C***H***_2_C = C-, *L6* C***H***_2_CO, *L7* C = CC***H***_2_C = C, *L8* -C***H***-, *EDTA* Ethylenediaminetetraacetic acid, *AS* Ankylosing spondylitis

Figure 1 shows typical ^1^H NMR spectra of the plasma urine and tissue samples taken from randomly selected participants in the AS and control groups. The urine, plasma and polar tissue extract samples contained mainly a series of amino acids, glucose and lipids, while the non-polar tissue extract samples contained the minimum metabolites, dominated mainly by triglycerides (TG).

### Multivariate data analysis of NMR data

**Fig. 2:**
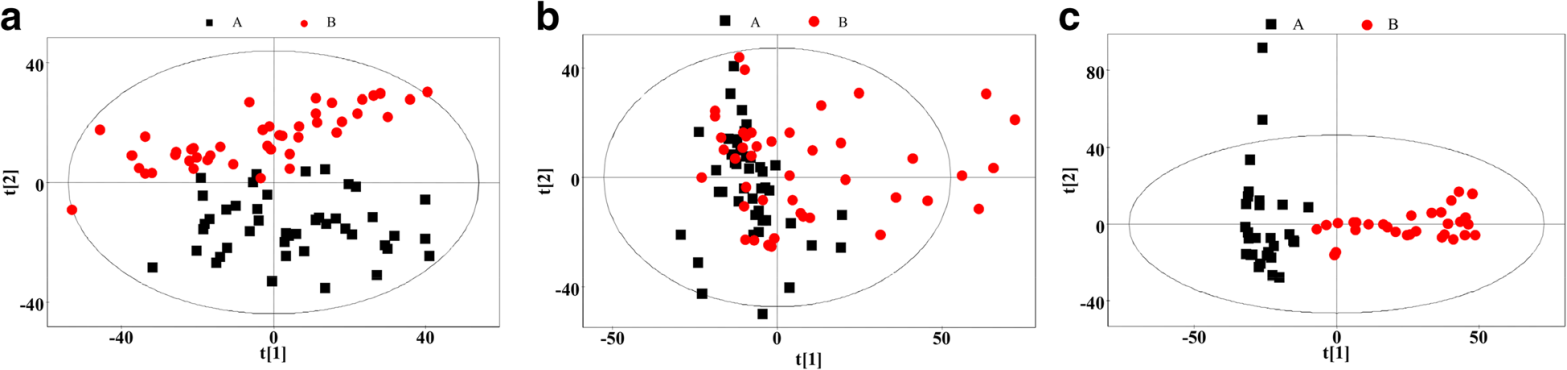
Principal component analysis (PCA) score plots based on t﻿he 1H NMR spectra of (**a**) plasma, (**b**) urine and (**c**) tissue metabolites obtained from the ankylosing spondylitis patients (AS, red circles) and healthy controls (HC, black squares). PC1 and PC2 explained (A-plasma) 34.5 % and 13.6 %, (B-urine) 31.5 % and 13.4 %, (C-tissue)48.2 % and 22.7 % of the variables, respectively

Figure 2 shows the PCA score plots for the three sample types (urine plasma and tissue). The difference in the PCA score plots of tissue samples between the AS group and the control group was the most significant difference. The PCA score plots of plasma samples revealed incomplete but clear discrimination between patients with AS and healthy control subjects. For the urine samples, the PCA model was not able to completely distinguish between the patients with AS and healthy control subjects. Therefore, we focused mainly on supervised analysis results for the urine samples

**Fig. 3:**
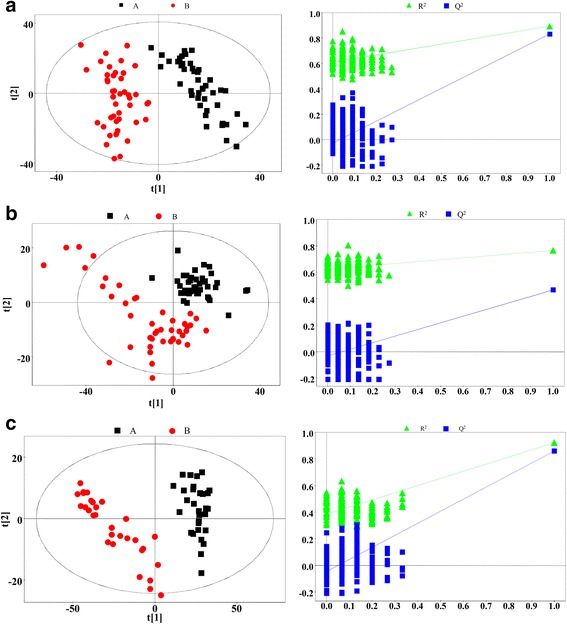
Partial least squares discriminant analysis (PLS-DA) score plots (*left panel*) and statistical validation of the corresponding PLS-DA model by permutation analysis (*right panel*) based on the ^1^H nuclear magnetic resonance spectra of (**a**) plasma, (**b**) urine and (**c**) tissue metabolites obtained from the patients with ankylosing spondylitis (*red circles*) and from the healthy control subjects and patients with femoral neck fracture (*black squares*). Principal component 1 (PC1) and PC2 explained (**a**) 42.1 % and 16.0 %, (**b**) 39.4 % and 11.3 %, and (**c**) 51.6 % and 18.7 % of the variables, respectively

The PLS-DA result is presented in Fig. 3. For these three types of samples, the score plots highlighted two clusters corresponding to the AS and control groups. In the PLS-DA model, the parameters of *R* ^2^ *Y* and *Q* ^2^ were, respectively, 0.894 and 0.832 (plasma), 0.767 and 0.469 (urine), and 0.921 and 0.860 (tissue extracts). These results revealed the good discrimination and predictive ability of this model. Although the *Q* ^2^ value of urine samples was slightly lower, it was acceptable considering the many uncontrollable factors in human studies. Model cross-validation through permutation tests (200 times) generated intercepts of *R* ^2^ and *Q* ^2^ (respectively, 0.599 and −0.029 for plasma, 0.617 and −0.027 for urine, and 0.385 and −0.047 for tissue). The low values of the intercepts indicate that the model was not over-fitted.

**Fig. 4:**
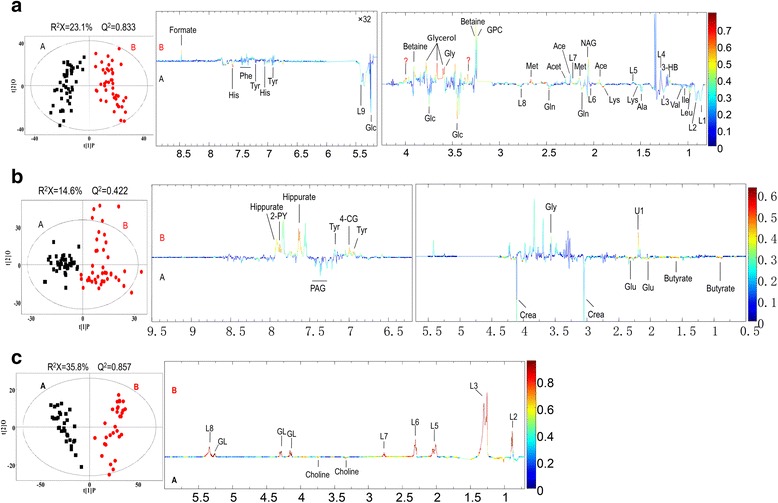
Orthogonal projection to latent structures discriminant analysis score plots (*left panel*) and the corresponding coefficient loading plots (*right panel*) based on the ^1^H nuclear magnetic resonance spectra of (**a**) plasma, (**b**) urine and (**c**) tissue metabolites obtained from the patients with ankylosing spondylitis (*red circles*) and from the healthy control subjects and patients with femoral neck fracture (*black squares*). *2-PY* 2-Pyridone-3-carboxamide, *4-CG* 4-Cresol glucuronide, *Ace* Acetate, *Ala* Alanine, *Crea* Creatinine, *GL* Glycerol of lipids, *Gln* Glutamine, *Glu* Glutamate, *Gly* Glycine, *GPC* Glycerophosphocholine, *His* Histidine, *Ile* Isoleucine, *L1* C***H***_3_(CH_2_)n., *L2* C***H***_3_CH_2_CH_2_C=, *L3* -(C***H***_2_)n-, *L4* C***H***_2_CH_2_CO, *L5* -C***H***_2_C = C-, *L6* C***H***_2_CO, *L7* C = CC***H***_2_C = C, *L8* -C***H*** = C***H***-, *Lac* Lactate, *Leu* Leucine, *Lys* Lysine, *Met* Methionine, *NAG* N-acetyl glycoprotein, *PAG* Phenylacetylglycine, *Phe* Phenylalanine, *Pyr* Pyruvate, *Tyr* Tyrosine, *Val* Valine

The OPLS-DA analytical results and the correlation coefficient loading plots are shown in Fig. 4. The parameters of *R* ^2^ *Y* and *Q* ^2^ were respectively, 0.894 and 0.833 (plasma), 0.767 and 0.422 (urine), and 0.921 and 0.857 (tissue extracts), which also revealed the good discrimination and predictive ability of this model. In addition, based on our results from the correlation coefficient loading plots, correlation coefficient cut-off values for potential markers of plasma, urine and tissue samples were set at 0.294, 0.294 and 0.355, respectively.

### Potential biomarkers and pathway analysis

**Fig. 5:**
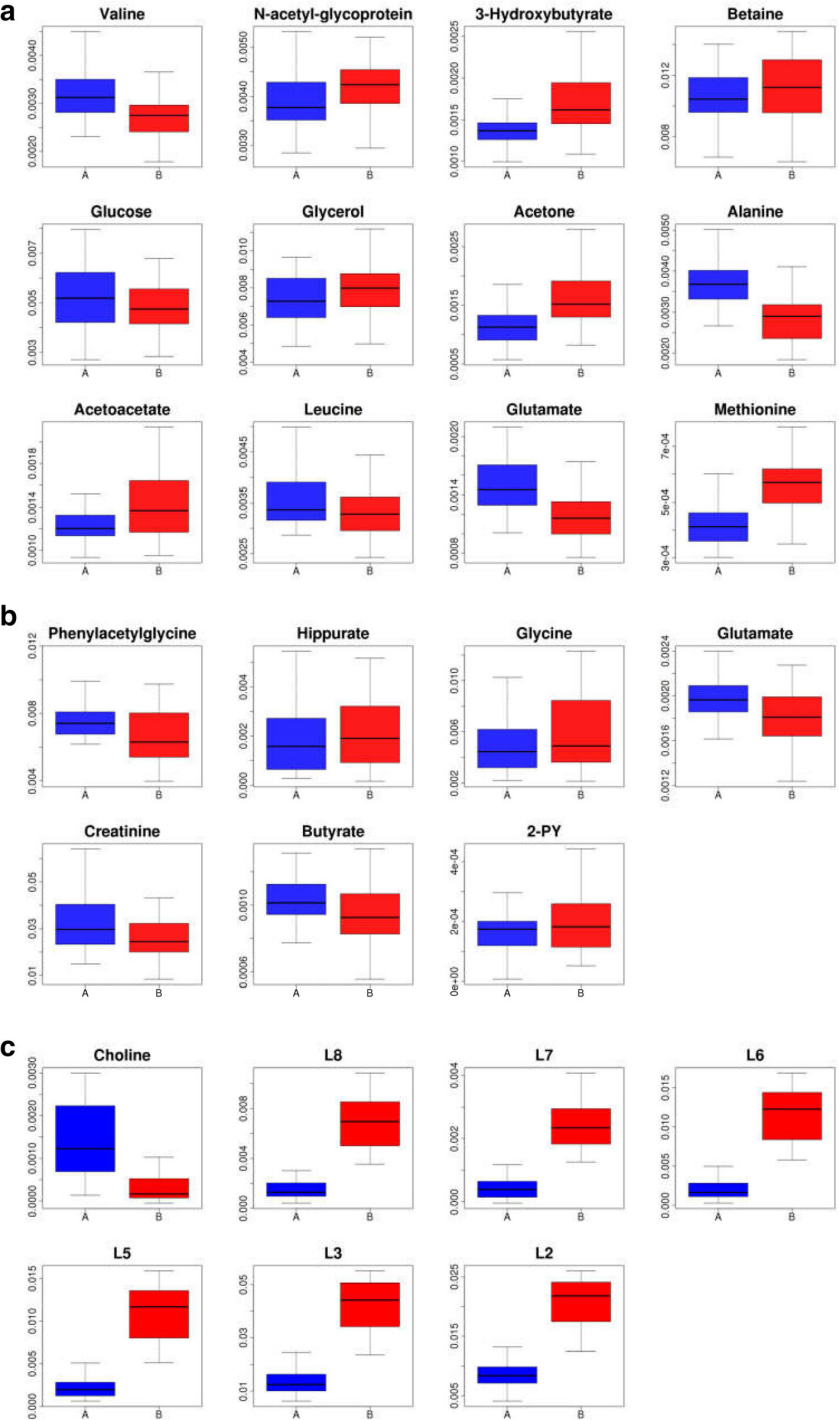
Box-and-whisker plots showing the relative levels of selected potential biomarkers for AS in (**a**) plasma, **b**) urine and **c**) tissue. *Horizontal line* in the middle portion of the boxes represents the median; *bottom* and *top boundaries of boxes* represent the lower and upper quartiles, respectively; and whiskers represent the 5th and 95th percentiles. *AS* Ankylosing spondylitis, *HC* Healthy control subjects, *FNF* Femoral neck fracture, *2-PY* 2-Pyridone-3-carboxamide, *L2* C***H***_3_CH_2_CH_2_C=, *L3* -(C***H***_2_)_n_-, *L5* -C***H***_2_C = C-, *L6* C***H***_2_CO, *L7* C = CC***H***_2_C = C, *L8* -C***H*** = C***H***-

**Fig. 6:**
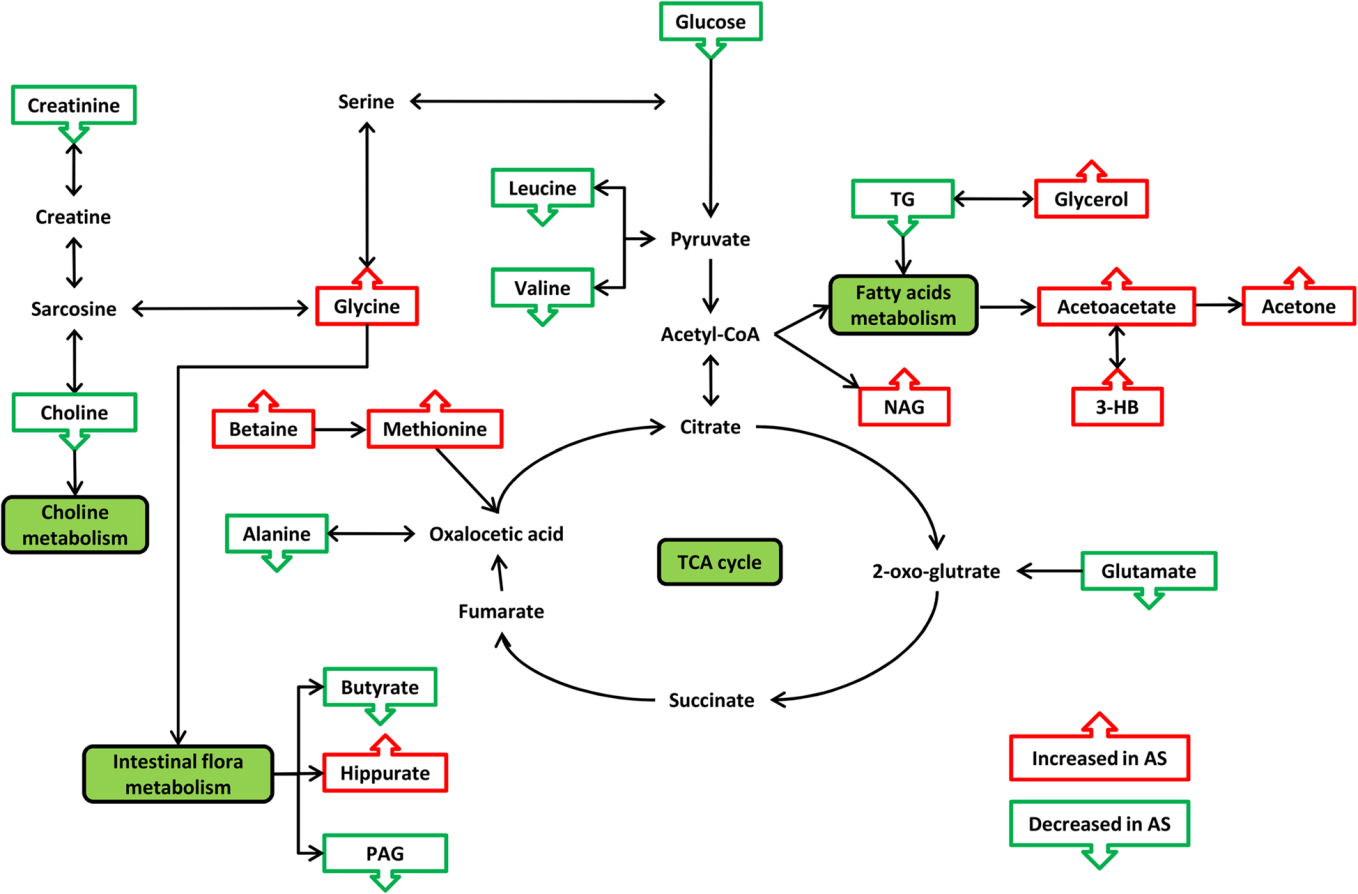
Altered metabolic pathways for the most relevant distinguishing metabolites (potential biomarkers) between the patients with AS and the healthy control subjects and patients with femoral neck fracture. *Green boxes* indicate metabolites that were up-regulated in AS, while *red boxes* indicate metabolites that were down-regulated. *AS* Ankylosing spondylitis, *3-HB* 3-Hydroxybutyrate, *NAG* N-acetyl glycoprotein, *TG* Triglycerides, *PAG* Phenylacetylglycine, *CoA* Coenzyme A, *TCA* Tricarboxylic acid

On the basis of correlation coefficients, VIP values and *P* values of the metabolites obtained from the multivariate analysis, we selected 20 metabolites from plasma (*n* = 13), urine (*n* = 7) and tissue (*n* = 2) as potential biomarkers for AS (Table 2). The box-and-whisker plots of these potential biomarkers also suggested the presence of significant intergroup differences (Fig. 5). On this basis, by KEGG pathway database retrieval, we found that these metabolites were associated mainly with metabolic pathways such as fat metabolism, intestinal microbial metabolism, glucose metabolism and choline metabolism, as well as probably with immune regulation. Finally, we combined these results to draw a metabolic pathway map (Fig. 6) to show a more intuitive correlation between these metabolites.

**Table 2:** Summary of potential biomarkers of ankylosing spondylitis by plasma, urine and tissue metabolomic analysis

Metabolite	Sample type	Status^a^	Chemical shift	Correlation coefficient (AS vs HC)^b^	VIP value^b^	FC^c^	*P* value^d^
Leucine	Plasma	↓	0.96 (d), 0.97 (d), 1.72 (m), 1.72 (m), 3.73 (t)	−0.452	0.698	0.932	0.045
Valine	Plasma	↓	1.00 (d), 1.05 (d), 2.28 (m), 3.62 (d)	−0.302	1.090	0.86	<0.001
3-HB	Plasma	↑	1.20 (d), 2.32 (dd), 2.42 (dd), 4.16 (m)	0.422	1.328	1.254	<0.001
Alanine	Plasma	↓	1.48 (d), 3.77 (q)	−0.332	1.664	0.770	<0.001
NAG	Plasma	↑	2.05 (s)	0.570	0.065	1.207	<0.001
Methionine	Plasma	↑	2.14 (s), 2.16 (m), 2.65 (t), 3.87 (t)	0.597	1.701	1.325	<0.001
TG (L1, L2, L3, L5, L7, L8)	Plasma	↓	0.86 (t), 0.88 (t), 1.27 (m), 2.01 (m), 2.76 (m), 5.30 (m)	−0.400	0.977	0.814–1.077	<0.05
Acetone	Plasma	↑	2.23 (s)	0.298	1.358	1.415	<0.001
Acetoacetate	Plasma	↑	2.29 (s)	2.294	0.884	1.249	<0.001
Betaine	Plasma	↑	3.26 (s), 3.91 (s)	0.334	0.287	1.038	0.030
Glycerol	Plasma	↑	3.65 (dd), 3.56 (dd), 3.77 (m)	0.328	1.400	1.207	<0.001
Glucose	Plasma	↓	3.42 (dd), 3.54 (dd), 3.71 (dd), 3.78 (m), 3.84 (m), 5.26 (d)	−0.317	0.559	0.909	0.013
Glutamate	Plasma	↓	2.07 (m), 2.35 (m), 3.75 (m)	−0.374	1.300	0.789	<0.001
Butyrate	Urine	↓	0.90 (t), 1.56 (m), 2.16 (t)	−0.447	1.533	0.896	0.003
Creatinine	Urine	↓	3.04 (s), 3.93 (s)	−0.331	1.330	0.814	0.018
Glycine	Urine	↑	3.56 (s)	3.392	1.088	1.298	0.009
Hippurate	Urine	↑	7.84 (d), 7.64 (t), 7.56 (dd)	0.421	0.772	1.320	0.017
PAG	Urine	↓	8.03 (d), 7.36 (m), 7.37 (m), 7.43 (m)	−0.306	1.213	0.895	0.028
Glutamate	Urine	↓	2.07 (m), 2.35 (m), 3.75 (m)	−0.429	1.541	0.919	0.002
2-PY	Urine	↑	7.83 (dd), 8.55 (d)	0.461	0.700	1.182	0.049
TG (L2, L3, L5, L6, L7, L8)	Tissue	↑	0.88 (t), 1.27 (m), 2.01 (m), 2.30 (m), 2.76 (m), 5.33 (m)	0.946	1.060	2.490–7.039	<0.001
Choline	Tissue	↓	4.14 (m), 4.29 (m), 5.26 (m)	−0.680	0.718	0.242	<0.001

*Abbreviations: AS* Ankylosing spondylitis, *VIP* Variable importance for projection, *FC* Fold change, *3-HB* 3-Hydroxybutyrate, *NAG* N-acetyl glycoprotein, *TG* Triglycerides, *PAG* Phenylacetylglycine, *2-PY* 2-Pyridone-3-carboxamide, *L1* C***H***_3_(CH_2_)_n_., *L2* C***H***_3_CH_2_CH_2_C=, *L3* -(C***H***_2_)_n_-, *L5* -C***H***_2_C = C-, *L6* C***H***_2_CO, *L7* C = CC***H***_2_C = C, *L8* -C***H*** = C***H***-

^a^Relative concentrations compared with healthy control subjects: ↑ = up-regulated; ↓ = down-regulated

^b^Correlation coefficient and VIP value were obtained from orthogonal projection to latent structures discriminant analysis

^c^Fold change between patients with AS and healthy control subjects

^d^ *P* value determined using Student’s *t* test

## Discussion

The diagnosis of AS is not prompt owing to the lack of effective diagnostic biomarkers. With effective and potentially disease-modifying treatments such as TNF inhibitors becoming widely available [31], the diagnostic delay becomes the critical rate-limiting factor for the mobility and quality of life of patients with AS. As an effective tool, metabolomics has been playing a great role in studies aimed at finding diagnostic biomarkers for various types of cancer [32]. In recent years, few studies have been reported on the metabolomics of AS [1, 2, 16], and the common point is that MS and only plasma samples are used in these studies. In the present study, for the first time to our knowledge, we used NMR along with plasma, urine and ligament tissues as samples in metabolomic investigations in an effort to define more meaningful potential diagnostic biomarkers for AS.

All metabolites with significant changes could be candidates for AS biomarkers (i.e. potential biomarkers). Nevertheless, no single biomarker can characterize AS, and fluctuating metabolic changes in amino acids are not specific biomarkers of any disease [29]. Thus, a panel of markers rather than a single compound may be promising tools for making an accurate diagnosis of AS [12]. On the basis of these detection methods and multivariate data analysis, we selected 20 different metabolites as potential biomarkers for AS.

The TG content was significantly decreased in the plasma of patients with AS while its metabolite level in plasma (glycerol) was significantly increased. More importantly, three metabolites of the β-oxidation of its downstream fatty acids—namely, acetoacetate, acetone and 3-hydroxybutyrate (3-HB)—were significantly increased in the plasma of patients with AS. In addition, two branched-chain amino acids (leucine and valine) were decreased in AS plasma. Branched-chain amino acids have long been thought to be closely related to fat metabolism, especially for leucine. It has been confirmed in relevant reports that lack of leucine can promote the metabolism of peripheral fats and energy [33]. Additionally, 2-pyridone-3-carboxamide (2-PY) is increased in AS urine, which is reported to be related to fat metabolism as well [34]. All of the above results suggest that the fat metabolic pathways may be active in AS. In addition, we have also reported in previous studies that the expression levels of some β-oxidation-related enzymes were up-regulated in AS, that is the active status of fat metabolism may be related to the pathogenesis of AS [23]. Moreover, Ottaviani et al. reported that BMI tends to increase during the course of AS treatment [35], implying that BMI values are partly related to the severity of AS. Syrbe et al. also reported that the serum levels of some adipokines (resistin and visfatin) were up-regulated in patients with AS, while serum resistin levels are related to markers of inflammation [36]. Gao et al. found that plasma levels of glycerol were increased in AS. Similarly, they suggested that the increased glycerol levels may result from the large in vivo fat consumption in patients with AS [8]. Therefore, we concluded that the fat metabolism in AS may be very active and that TG, glycerol, acetoacetate, acetone, 3-HB, leucine, valine and 2-PY are the key metabolites reflecting these abnormalities. It is worth noting that TG content in the AS ligament tissue increased dramatically instead (Fig. 5). We think this may be an ectopic fat deposition phenomenon. It has been found in some studies that patients with fat deposition in local tissue often also have an increased level of inflammatory cytokines (such as TNF-α and interleukin 6) [37, 38], which is consistent with the chronic inflammatory environment in patients with AS.

IBD has been considered one of the most extra-articular symptoms of AS [39] and the pathogenesis of IBD is closely related to intestinal microorganisms [40]. Four of the potential biomarkers we detected are closely related to intestinal microbial metabolism. The levels of glycine and hippurate were significantly increased in AS urine. Hippurate is produced in the gut by microorganisms using glycine and benzoic acid as building blocks [41]. It is normally found in urine, and an increased level of hippurate indicates disorder of the gut microbiota [42]. In addition, an increased hippurate level in the urine has been associated with leanness [43], which is also consistent with our results related to abnormal fat metabolism. Phenylacetylglycine (PAG) and butyrate are also decreased in AS urine. Butyrate has been considered to regulate the intestinal flora balance, and the addition of low doses of butyrate to the diet has a certain therapeutic effect on ulcerative colitis [44]. On one hand, a decreased level of PAG in the urine may also be associated with intestinal flora metabolism disorder [45]. On the other hand, previous studies have found that sulphate-reducing bacteria were increased in the faeces of patients with AS, and such bacteria were suggested to be correlated with the pathogenic mechanism of IBD [46]. In addition, some authors believe that the intestinal microbial composition in patients with AS is changed compared with that in healthy individuals [47, 48], and *Dialister* has been suggested as the potential marker of AS [48]. Therefore, the evidence described above suggests that the changes of butyrate, hippurate, glycine and PAG levels in the urine may indicate that they are the key metabolites of the abnormal intestinal microbial metabolism in AS.

In our previous research we reported that insulin receptor (INSR) may be a key upstream protein that leads to an unusually active fat metabolism [12] and that over-expressed INSR can enhance the action of insulin and disrupt glucose metabolism. In this study, plasma glucose was decreased, which might be a result of the over-expression of INSR. In addition, as two important precursors of gluconeogenesis, alanine and glutamate were significantly decreased in AS plasma (glutamate was reduced in urine, too), which may also be the reason for the decline in the glucose concentration.

We also found abnormal choline metabolism in AS. Choline was decreased significantly in the ligaments of patients with AS. Choline has an affinity for fat and can promote the transport of fat out of the liver in the form of phospholipids through the blood or it can improve the use of fatty acids themselves in the liver to prevent the abnormal deposition of fat in some organs or tissues [49]. Therefore, the ectopic fat deposition phenomenon in the ligament may be related to the significant decrease in choline. Furthermore, as a precursor of choline, the urinary creatinine level in patients with AS was decreased. A decreased level of urinary creatinine is commonly found in chronic kidney disease, and AS is occasionally complicated with renal disease [50]. Therefore, the decreased urinary creatinine may be a manifestation of AS complicated with extra-articular symptoms.

AS is a typical autoimmune disease and its pathogenesis is closely related to immune system disorder. We found that plasma levels of betaine, methionine and N-acetyl glycoprotein (NAG) were noticeably increased. Methionine is a precursor of methionine enkephalin (MENK), and the latter is involved in the regulation of immune response in addition to affecting cell proliferation. Effects of MENK on the immune system are observed mainly in immune stimulation at lower concentrations [51]. Thus, an elevated plasma level of methionine may reflect reduced synthesis of MENK, which further results in an aggressive autoimmune reaction leading to AS. Because betaine is a precursor of methionine [52], the elevation of both betaine and methionine in AS may be inter-related, and plasma NAG elevation may also suggest an immune system disorder in AS because NAG can acetylate glycoproteins, which are important for white blood cell recognition [53].

In general, the metabolomic profiles we have obtained are promising. With good discrimination and predictive ability in multivariate analysis models, the profiles could aid in making decisions on a more invasive diagnostic procedure. However, some limitations of this research need to be noted. First, the sample was limited in size, and the results should be validated with a larger number of patients with AS in the future. Second, the disease activity in patients with AS was not evaluated, and further work is required to address the relationship between potential biomarkers and disease activity. Third, some comorbidities of AS may affect the patient’s own metabolic status, although we excluded this category of patients in advance. However, considering the possible existence of bias, we still need to pay attention to this problem when interpreting the results. Fourth, the medication history of the patients, which may also influence the results of metabolic profiling, was not interpreted in our results.

## Conclusions

In this study, NMR along with three sample types—urine, plasma and ligament tissue—were used for the first time in a metabolomic study of AS to obtain more comprehensive metabolomic profiles and later select potential biomarkers that may help decrease the delay in the diagnosis of AS. Disorders of four metabolic pathways as well as immune function may exist in patients with AS, and 20 differential metabolites that play critical roles in these metabolic pathways or physiological functions can be considered as potential biomarkers for AS. Further validation studies are needed to confirm these results before this method can be transferred from bench to bedside.

## Abbreviations

2-PY: 2-Pyridone-3-carboxamide
3-HB: 3-Hydroxybutyrate
4-CG: 4-Cresol glucuronide
Ace: Acetate
Ala: Alanine
AS: Ankylosing spondylitis
BASDAI: Bath Ankylosing Spondylitis Disease Activity Index
BASFI: Bath Ankylosing Spondylitis Functional Index
BMI: Body mass index
Citr: Citrate
CoA: Coenzyme A
EDTA: Ethylenediaminetetraacetic acid
EP: Epoxy epoxide
FC: Fold change
FNF: Femoral neck fracture
GL: Glycerol of lipids
Gln: Glutamine
Glu: Glutamate
Gly: Glycine
GPC: Glycerophosphocholine
HC: Healthy control subjects
His: Histidine
IBD: Inflammatory bowel disease
Ig: Immunoglobulin
Ile: Isoleucine
INSR: Insulin receptor
KEGG: Kyoto Encyclopedia of Genes and Genomes
L1: CH_3_(CH_2_)n.
L2: CH_3_CH_2_CH_2_C=
L3: -(CH_2_)n-
L4: CH_2_CH_2_CO
L5: -CH_2_C = C-
L6: CH_2_CO
L7: C = CCH_2_C = C
L8: -CH = CH-.
Lac: Lactate
Leu: Leucine
Lys: Lysine
MENK: Methionine encephalin
Met: Methionine
MS: Mass spectrometry
NAG: N-acetyl glycoprotein
NMR: Nuclear magnetic resonance
OAG: O-acetyl glycoprotein
OPLS-DA: Orthogonal projection to latent structures discriminant analysis
PAG: Phenylacetylglycine
PC: Phosphorylcholine
PCA: Principal component analysis
Phe: Phenylalanine
PLS-DA: Partial least squares discriminant analysis
Pyr: Pyruvate
RA: Rheumatoid arthritis
TCA: Tricarboxylic acid
TG: Triglycerides
*t*_m_: Mixing time
TMSP: 3-Trimethylsilyl-2,2,3,3-d_4_-propionate
TNF-α: Tumour necrosis factor α
Tyr: Tyrosine
Val: Valine
VIP: Variable importance for projection
